# Physiological response mechanism of heavy metal‐resistant endophytic fungi isolated from the roots of *Polygonatum kingianum*

**DOI:** 10.1111/1758-2229.13194

**Published:** 2023-08-21

**Authors:** Guan‐Hua Cao, Xiao‐Gang Li, Chen‐Rui Zhang, Yi‐Ran Xiong, Xue Li, Tong Li, Sen He, Zheng‐Guo Cui, Jie Yu

**Affiliations:** ^1^ School of Chinese Materia Medica Yunnan University of Chinese Medicine Kunming China; ^2^ Department of Environmental Health University of Fukui School of Medical Sciences Fukui Japan

## Abstract

This study aims to evaluate the tolerance of endophytic fungi isolated from the fibrous roots of *Polygonatum kingianum* to arsenic (As) and cadmium (Cd) and their physiological response mechanisms. Five isolated strains were obtained with EC_50_ values for As(V) ranging from 421 to 1281 mg/L, while the other three strains tolerated Cd(II) with an EC_50_ range of 407–1112 mg/L. Morphological and molecular identification indicated that these eight strains were *Cladosporium* spp. belonging to dark septate endophytes (DSEs)*.* The contents of metal ions in mycelium sharply increased, reaching 38.87 mg/kg for strain MZ‐11 under As(V) stress and 0.33 mg/kg for fungus PR‐2 under Cd(II). The physiological response revealed that the biomass decreased with increasing concentrations of As(V) or Cd(II), and the activity of superoxide dismutase significantly improved under the corresponding EC_50_‐concentration As/Cd of the strains, as well as the contents of antioxidant substances, including metallothionein, glutathione, malondialdehyde, melanin, and proline. Taken together, the filamentous fungi of *Cladosporium* spp. accounted for a high proportion of fungi isolated from the fibrous roots of *P. kingianum* and had a strong capacity to tolerate As(V) or Cd(II) stress by improving antioxidase activities and the content of antioxidant substances, and immobilization of metal ions in hyphae.

## Graphical abstract

Some DSEs isolated from the fibrous roots of *P. kingianum* had a strong capacity to tolerate As(V) or Cd(II) stress by improving antioxidase activities and the contents of antioxidant substances, and immobilization of metal ions in cell walls, thus scavenging free radical and reducing oxidative damage.

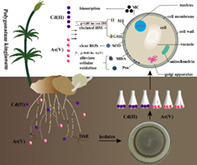

## INTRODUCTION

*Polygonatum kingianum* Coll. et Hemsl belongs to the genus *Polygonatum* and has tremendous therapeutic value. The rhizome, the main medicinal part of *P. kingianum*, is effective in treating fatigue, weakness, cough, and hyperlipidemia, as well as in preventing diabetes (Gu et al., 2020; Yan et al., 2017; Zhao et al., 2018). The medicinal plant is mainly cultivated in Yunnan Province, where the soil is contaminated by heavy metals (HMs), such as cadmium (Cd), arsenic (As), and lead (Pb) (Zhang et al., 2014). Therefore, it poses a formidable threat to the growth and medicinal safety of *P. kingianum* (Wang et al., 2019).

The toxicity of Cd and As threatens human life and health through the food chain, causing acute and chronic poisoning (Mishra et al., 2019). Cd causes growth inhibition and possible death in medicinal plants and can be enriched in plants and enter the food chain, causing a series of hazards to human health (Al‐Khayri et al., 2023). The toxicity and carcinogenic properties of As can contribute to the production of reactive oxygen species and the inactivation of functional proteins in plants, which are undeniable once crops or medicinal plants with an excessive accumulation of As are consumed by humans (Allevato et al., 2019). Increased risk of Cd and As contamination in herbal medicines has been reported (Wang et al., 2019; Yang et al., 2021), indicating that it is imperative to find a safe, effective, environmentally friendly, and sustainable method to address the harmful effects of Cd/As accumulation in medicinal herbs and crops.

Plant endophytic fungi are considered natural candidates to improve tolerance and decrease the accumulation of HMs. Dark septate endophytes (DSEs) are a class of endophytic fungi with typical characteristics of dark mycelia and diaphragmatic hyphae and play positive roles in nutrient uptake and HM resistance. Many studies have shown that DSE strains widely colonize plant roots in HM stress habitats (Berthelot et al., 2016). Previous studies have shown that the DSE fungus *Exophiala pisciphila* H93 can activate antioxidant systems and alter chemical forms and translocations, thus reducing the phytotoxicity of Cd and promoting maize growth (Wang et al., 2016). *Humicola* sp. reduces As toxicity and improves the growth of *Bacopa monnieri*, mainly through As biomethylation in the soil (Tripathi et al., 2020).

According to existing studies, the resistance capacity of DSE to HM stress is mainly achieved by biosorption, bioaccumulation, antioxidant response, and efflux of metal ions (Priyadarshini et al., 2021). Melanin is a common compound in the cell walls or spores of DSEs and is involved in HM biosorption via carboxyl and hydroxyl groups (Berthelot et al., 2020). Metallothionein (MT) and reduced glutathione (GSH) are key factors in HM accumulation and detoxification in DSE strains by fixing metal ions and subsequently sequestering them in vacuoles (Banerjee et al., 2018; Robinson et al., 2021). In addition, DSE strains commonly decrease oxidative damage to strengthen HM resistance by improving the activity of antioxidant enzymes and the contents of antioxidant substances such as superoxide dismutase (SOD), proline (Pro), and malondialdehyde (MDA) (Hou et al., 2020).

Few studies have focused on the use of ‘dominant DSE‐medicinal plant’ combinations to alleviate HM contamination in medicinal plants, particularly in *P. kingianum*. In the present study, Cd/As‐resistant DSE strains were screened from the roots of *P. kingianum*, and their taxonomic status and physiological response mechanisms were uncovered. The results obtained will be helpful for soil bioremediation and ecological planting of herbal plants in HM‐containing areas.

## MATERIALS AND METHODS

### Material collection of plant samples

Healthy *P. kingianum* roots were sampled from Mangshi Prefecture Yunnan Province China in September 2019. The Cd and As concentrations were in the range of 0–0.68 and 0.04–52.18 mg/kg in the surrounding soil, respectively (Zhang et al., 2014). After sampling, the fibrous roots and tubers were placed in plastic bags containing ice packs and immediately returned to the laboratory. The root materials were treated within 3 days, and the remaining samples were stored at −80°C for standby application.

### Isolation of endophytic fungi from roots

Surface disinfection and isolation of endophytic fungi were performed according to the procedures described by Zheng et al. (2017). The root samples were repeatedly rinsed with running water to remove adhering soil particles and microorganisms until the water was not turbid. The fibrous roots were then soaked in a 75% ethanol solution for 3–4 min, transferred to a 5% (w/v) sodium hypochlorite solution for 5–7 min, and rinsed three times with sterile distilled water. Subsequently, roots were cut into 1.5 cm long segments with a sterile scalpel and placed in potato dextrose agar (PDA) and malt extract agar (MEA) media containing 1% penicillin–streptomycin. The incubation conditions for endophytic fungi in root segments were set at 28 ± 1°C without illumination. Colonies gradually emerged around the root segments from the third day, and each colony was then transferred to an antibiotic‐free PDA medium for isolation and purification. Before tolerance tests of strains to HM, the purified strains were stored in the test tubes filled with a slant PDA medium at 4°C.

### Tolerance of isolated strains to As(V) and Cd(II)

Tolerance of the isolated strains to As(V) and Cd(II) was determined by measuring their biomass at different metal ion concentrations. First, the strains were activated on PDA plates for 2 weeks. Six blocks of 0.7 cm in diameter were cut from the edge of the colony and transferred to 100 mL of modified Melin‐Norkrans (MMN) medium (20 g of glucose, 0.15 g of MgSO_4_, 0.025 g of NaCl, 0.05 g of CaCl_2_, 1.2 mL of FeCl_3_ (1%), 0.5 g of KH_2_PO_4_, 0.25 g of (NH_4_)_2_HPO_4_, 100 μg of vitamin B_1_, and adding distilled water to obtain 1000 mL of solution, pH 5.8) containing 100 mg/L As(V) or Cd(II) for culturing at 28 ± 1°C and 180 rpm for 1 week (Cao et al., 2019). Strains that grew well in 100 mg/L As(V) or Cd(II) were selected as the target strains for further study. Target strains were cultured in MMN media with gradient concentrations of As(V) (0, 100, 200/250, 400, 600, 800, 1200, and 1800 mg/L) or Cd(II) (0, 100, 200, 400, 800 and 1200 mg/L), of which the dry weight was determined by drying at 70°C until constant weight (Diao et al., 2013). There were five replicates for each concentration. After culturing, the effective concentration (EC_50_) value was generated by fitting a linear regression to the results of biomass inhibition for each strain, and the EC_50_ was used to evaluate the HM tolerance of the test strains (Pauliina et al., 2011).

### Morphological and molecular identification of endophytic fungi

The morphological and micromorphological features of these fungi were determined using pure and coverslip‐inserted cultures in a PDA medium, as described by Bensch et al. (2012). Micromorphological features of the reproductive structures of the isolated strains on each coverslip were observed under a light microscope of ZEISS Axio Scope. A1 (Carl Zeiss AG, Germany), and images were used to distinguish DSE from non‐DSE, as described by Sieber and Grünig (2013) and Rodriguez et al. (2009).

Molecular identification of target fungi was performed by polymerase chain reaction (PCR) amplification using the universal primer pairs ITS1 (5′‐TCCGTAGGTGAACCTGCGG‐3′) and ITS4 (5′‐TCCTCCGCTTATTGATATGC‐3′) (Gharsallah et al., 2020). First, genomic DNA was extracted from mycelia cultured in an MMN liquid medium for 7 days according to the instructions of the Microbial DNA Extraction Kit (Bioteke, China). The PCR mixture consisted of 2 μL of genomic DNA template, 9 μL of 2× PCR Master Mix (Fermentase, USA), 1 μL of forward primer, 1 μL of reverse primer, and 12 μL of water in a total volume of 25 μL. The PCR cycling protocol consisted of initial denaturation at 94°C for 4 min, followed by 30 cycles of 94°C for 45 s, 55°C for 45 s, and 72°C for 1 min. The final extension step was 72°C for 10 min. The amplified DNA was sequenced by Tsingke Biotechnology Company (China), and a sequence similarity search was performed using BLAST in NCBI (http://blast.ncbi.nlm.nih.gov). Homologous fungal internal transcribed spacer (ITS) regions were retrieved from NCBI, and a phylogenetic tree was constructed using the neighbour‐joining method with 1000 bootstrap retests using MEGA software version 7.

### Biosorption and accumulation of total As and Cd by DSE strains

After determining the EC_50_ values of the DSE strains, the biosorption and accumulation of total Cd and As in the mycelia were evaluated. Total mycelium of DSE strains was collected by suction filtration after culturing for 1 week at 28°C in 100 mL MMN medium, of which the mycelium of each triangular flask was rinsed thoroughly three times with 25 mL of 10 mmol/L EDTA to remove attached metal ions (Cao et al., 2019). For the total As, a dried mycelium sample of 0.100–0.300 g for each treatment group was digested with mixed acid consisting of nitric acid, perchloric acid, and concentrated sulfuric acid (5:1.5:1) at 220°C for 6–8 h, and the digestion solution was evaluated using a PF6 atomic fluorescence spectrophotometer (Persee, China) according to the manufacturer's instructions. For total Cd, 0.300–0.500 g of dried mycelium was digested in a mixture of nitric acid and perchloric acid (9:1). The resulting filtrate was transferred to a 25 mL volumetric flask and diluted to scale with nitric acid (1%) (Norouzi et al., 2012). The total Cd content of the DSE strains was determined using an AA660 flame atomic absorption spectrophotometer (Shimadzu, Japan).

### Antioxidant system of target DSE strains

The activities of antioxidant enzymes and the contents of antioxidant materials were determined to analyse the physiological response mechanism of the target DSE strains to Cd(II)/As(V). Target DSE strains were cultured in 100 mL MMN medium triangular flasks with EC_50_ concentrations of As(V) or Cd(II) at 28 ± 1°C and 120 rpm for 7 days. Mycelia were collected to determine each antioxidant index.

A 1.0 g sample of mycelium was weighed and ground in an ice bath with 4 mL of phosphate buffer and centrifuged at 12,000×*g* and 4°C for 10 min. The supernatant was used to determine the SOD activity at 325 nm using the classical autoxidation method (Li, 2012). MT and melanin content were determined using an enzyme‐linked immunosorbent assay (ELISA) kit (Meimian Technology, China). GSH, MDA, and Pro concentrations were determined using the corresponding quantitative assay kits (SinoBestBio, China).

### Statistical analysis

Statistical analysis was performed using SPSS 24.0 and GraphPad 9.0 software. Assumptions of normality and homogeneity of variance were tested prior to all statistical tests. Tukey's honestly significant difference (HSD) test of one‐way analysis of variance (ANOVA) was used to analyse the differences between more than two groups or levels under the same variable condition at a level of 0.05, as shown in Figures 2, 4, and 5. An independent‐sample *t*‐test was used to determine the difference between the treatment and control groups (Figure 5) at levels of 0.05, 0.01, and 0.0001. All values are reported as means ± standard error (SE) (*n* = 3). Graphical analysis was performed using the Origin 2021 software package.

## RESULTS

### Morphological characteristics of isolated strains

**FIGURE 1:**
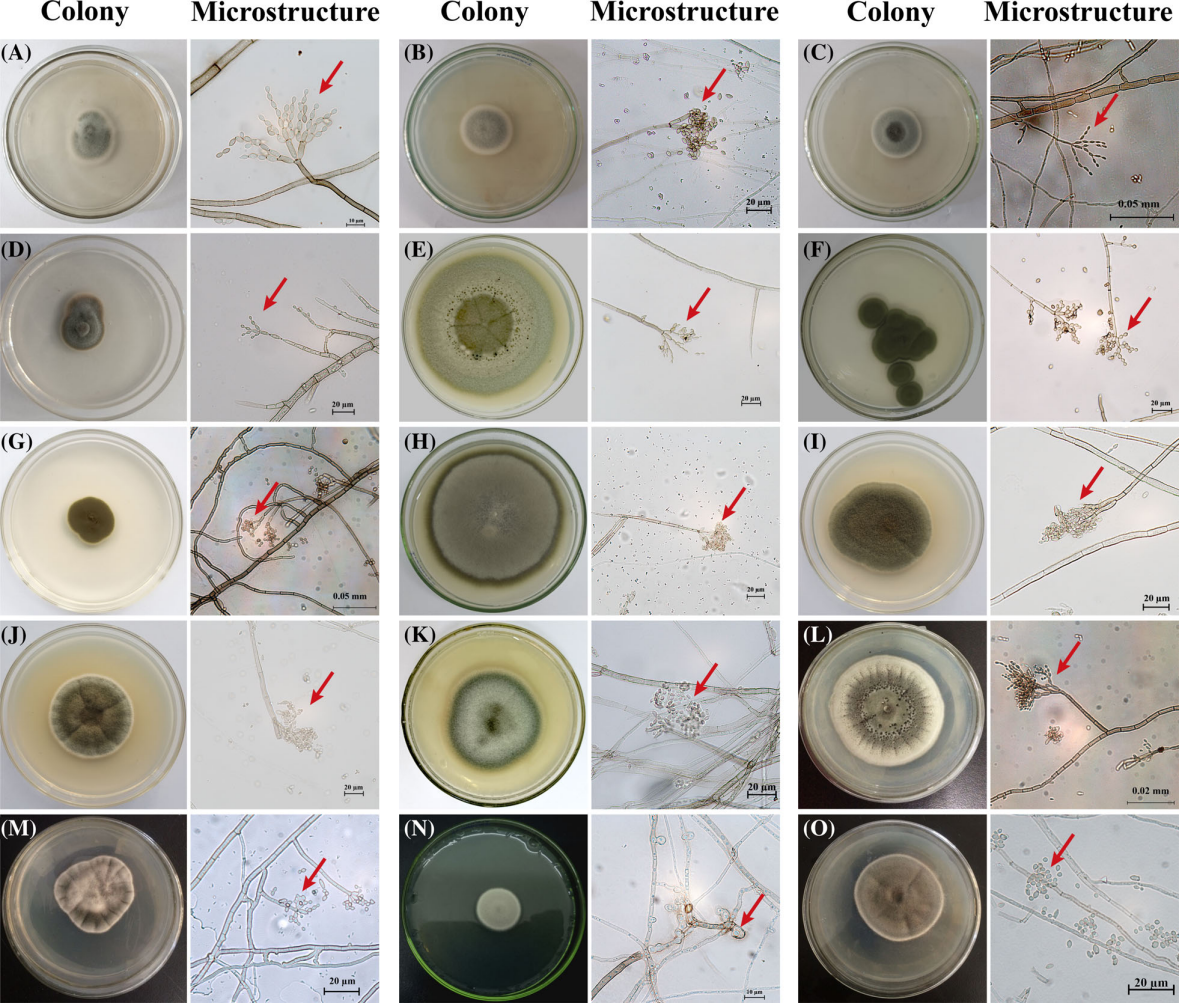
Morphology of 15 dark septate endophyte (DSE) colonies on potato dextrose agar medium (left) and their microstructure (right). The 15 DSE strains' hyphae had diaphragms, and there were varying numbers of conidia distributed around them. The 15 colonies were generally dark in colour, and the texture was compact. A, HXC‐5; B, MZ‐6; C, HLX; D, HG‐2; E, MZ‐1; F, DT‐7; G, DT‐5; H, MZ‐11; I, PR‐2; J, PR‐3; K, GJ‐12; L, MZ‐2; M, CB‐10; N, MZ‐4; O, MZ‐12.

A total of 23 endophytic fungi were isolated from the roots of *P. kingianum*, of which 15 were classified as DSE according to their morphological characteristics. As shown in Figure 1, the hyphae of DSE strains with diaphragms grew outward in an almost straight line and finally formed round or oval colonies. In addition, significant differences were observed in the growth rates of these strains. Strains of MZ‐11 and GJ‐12 were capable of growing on almost the entire plate after culturing for 7 days, whereas colonies of the other strains occupied only 1/3–1/2 of the plate. Colony edges changed from pale black to dark brown over time. The microstructural results revealed that the 15 DSE strain hyphae had diaphragms, and there were varying numbers of conidia distributed around them. The detailed morphological characteristics are summarized in Table 1.

**TABLE 1:** Morphological and cultural characteristics of 15 dark septate endophyte strains.

	Colony colour	Colony texture	Conidiophore	Conidia
HXC‐5	Grey olivaceous in the centre, margins white to grey olivaceous	The centre is raised, and the margin is flat, with aerial mycelia diffuse, reverse olivaceous black	Straight, solitary, branched, terminal or lateral, and without nodules	Limoniform, ovoid, obovoid to subglobose, aseptate
MZ‐6	Grey‐olivaceous in the centre, margins white to grey‐olivaceous	The centre is raised, and the margin is flat, with aerial mycelia diffuse, reverse olivaceous black	Straight, solitary, branched, terminal or lateral, without nodules	Ovoid, obovoid to subglobose, aseptate
HLX	Grey olivaceous in the centre, margins white to grey‐olivaceous	Concave in the centre, flat at the margins, with aerial mycelia diffuse, reverse olivaceous black	Straight, solitary, unbranched, terminal or lateral, and without nodules	Limoniform
HG‐2	Dark brown in the centre, margins grey to grey–brown	Concave in the centre, flat at the margins, with aerial mycelia diffuse, reverse dark brown	Straight, solitary, unbranched, terminal or lateral, and without nodules	Limoniform or subglobose
MZ‐1	Olivaceous in the centre; grey to olivaceous for margins	The centre is raised, and the margin is flat, with aerial mycelia diffuse, reverse olivaceous	Straight, solitary, unbranched, terminal or lateral, and without nodules	Limoniform or subglobose
DT‐7	Dark olivaceous from centre to margin	Flat, powdery, reverse dark olivaceous	Straight, solitary, unbranched, terminal or lateral, and without nodules	Globose, limoniform
DT‐5	Dark olivaceous from centre to margin	Flat, powdery, reverse dark olivaceous	Straight, solitary, unbranched, terminal or lateral, and without nodules	Globose
MZ‐11	Grey olivaceous from centre to margin	Flat with aerial mycelia diffuse, reverse dark olivaceous	Straight, solitary, unbranched, terminal or lateral, and without nodules	Limoniform, ovoid, obovoid to subglobose
PR‐2	Grey olivaceous from centre to margin	Bump on the margins and aerial mycelia diffuse, reverse dark olivaceous	Straight, solitary, unbranched, terminal or lateral, and without nodules	Limoniform, ovoid, obovoid to subglobose
PR‐3	Olivaceous‐black in the centre, off‐white at the margins	Concave in the centre, raised at the margins, and aerial mycelia diffuse, reverse dark olivaceous	Straight, solitary, unbranched, terminal or lateral, and without nodules	Globose or subglobose
GJ‐2	Grey olivaceous in the centre, white to grey olivaceous in margins	Concave in the centre, raised at the margins, and aerial mycelia diffuse, reverse olivaceous	Straight, solitary, unbranched, terminal or lateral, and without nodules	Limoniform or subglobose
MZ‐2	Olivaceous black–grey–white from centre to margin	Concave in the centre, flat at the margins, with aerial, floccose‐felty, dark olivaceous for reverse side; water droplets are produced on the surface of the mycelium	Straight, solitary, unbranched, terminal or lateral, and without nodules	Limoniform, ovoid, obovoid to subglobose, aseptate
CB‐10	Grey in the centre, grey–black at the margins	Concave in the centre, raised at the margins, and aerial mycelia diffuse, dark on the reverse side	Straight, solitary, unbranched, terminal or lateral, and without nodules	Globose or subglobose
MZ‐4	Grey in the centre and margins	The centre is raised, the margin is flat, with aerial mycelia diffuse, reverse side grey–black	Sorus	Limoniform or subglobose
MZ‐12	Grey–black–white from centre to margin	Concave in the centre, flat at the margins, with aerial mycelia diffuse, dark on the reverse side	Straight, solitary, unbranched, terminal or lateral, and without nodules	Limoniform or subglobose

### Tolerance of isolated strains to As(V) or Cd(II)

#### Screening of As/Cd‐resistant strains

**FIGURE 2:**
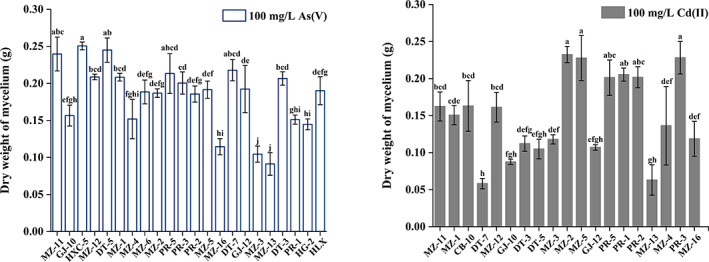
Dry weight of mycelium cultured in 100 mL modified Melin‐Norkrans medium containing 100 mg/L As(V) (left) or Cd(II) (right). Five strains of MZ‐11, DT‐5, HXC‐5, HLX, and HG‐2 had high As tolerance, while strains MZ‐2, PR‐3, and PR‐2 showed strong Cd‐resistant ability. Different lowercase characters indicate statistically significant differences among strains under the treatment of As(V) or Cd(II), *p* ≤ 0.05.

A total of 23 test isolates (15 DSE strains and 8 non‐DSE strains) were screened for strains with HM tolerance in 100 mL of MMN medium containing 100 mg/L Cd(II) or As(V), respectively. In vitro, the dry weight of the mycelia decreased with the addition of Cd(II) or As(V). As demonstrated in Figure 2, the strains with higher dry mycelial weight (>0.20 g) accounted for 39.13% under As(V) stress, for example, 0.25 g of HXC‐5, 0.24 g of DT‐5, and 0.24 g of MZ‐11. For HG‐2, the addition of As noticeably reduced growth and decreased biomass.

Most strains showed sensitivity to 100 mg/L Cd(II), and 68.72% of strains had a biomass of less than 0.20 g. However, strains of MZ‐2, PR‐3, and PR‐2 possessed a strong capacity for propagation under Cd(II) stress, and the corresponding dry weights were 0.22, 0.20, and 0.21 g, respectively.

Taken together, the tolerance of the strains to As(V)/Cd(II) showed obvious differences. Five strains, MZ‐11, DT‐5, HXC‐5, HLX, and HG‐2, were selected as target fungi to study their resistance mechanisms under As(V) stress, and strains MZ‐2, PR‐3, and PR‐2 under Cd(II) stress.

#### Effects of different As(V)/Cd(II) concentrations on the biomass of resistant strains

**FIGURE 3:**
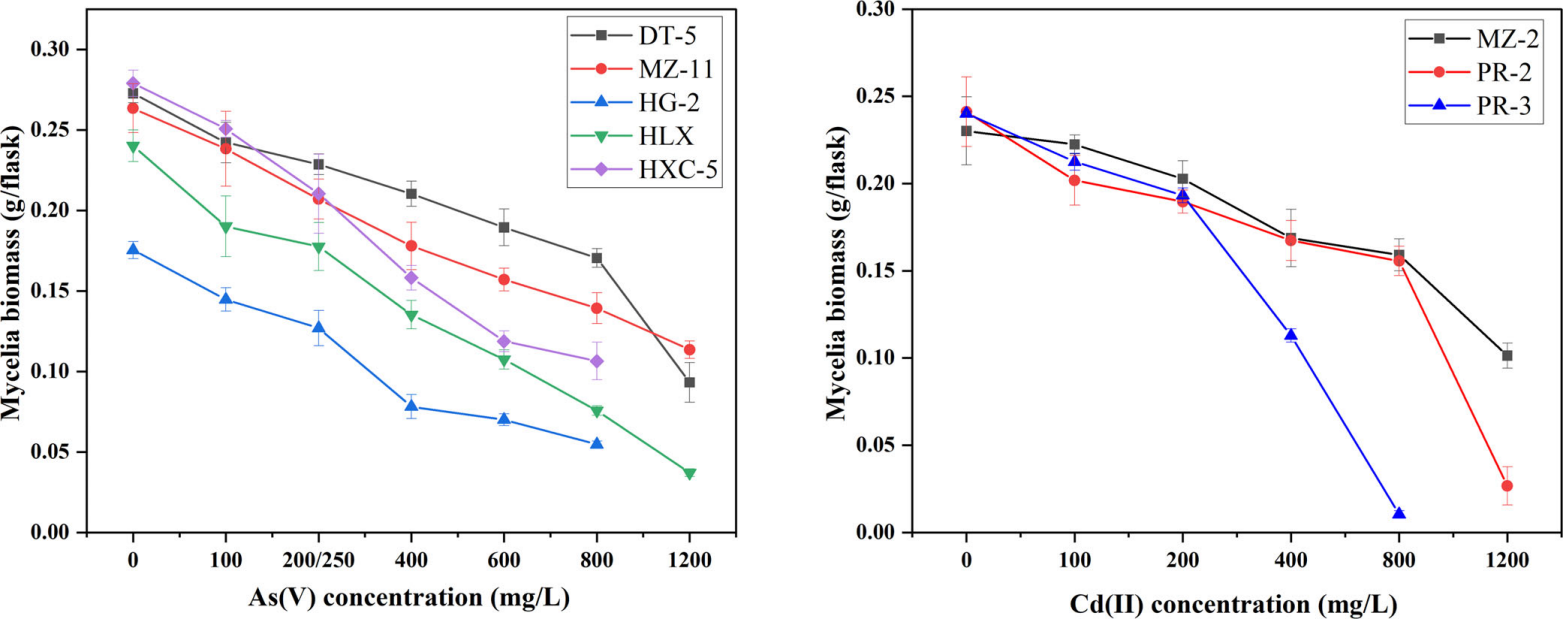
Dry weight of mycelium cultured in 100 mL modified Melin‐Norkrans medium supplement with different concentrations of As(V) or Cd(II) for 1 week. Elevated Cd(II) and As(V) concentrations led to a reduction in mycelial biomass with a linear phase‐off in a certain range, and EC_50_ values were calculated by fitting linear regressions to the biomass inhibition results. Strains of DT‐5 and MZ‐11 had higher tolerance to As(V), with EC_50_ values of 1281 and 1108 mg/L, and the EC_50_ values of strains MZ‐2 and PR‐2 were 1112 and 1104 mg/L, respectively, showing high Cd tolerance. As(V): 0, 100, 200/250, 400, 600, 800, and 1200 mg/L; Cd(II): 0, 100, 200, 400, 800, and 1200 mg/L.

Elevated Cd(II) and As(V) concentrations led to a reduction in mycelial biomass with a linear phase‐off within a certain range (Figure 3). The EC_50_ values were calculated by fitting linear regressions to the biomass inhibition results. Strains DT‐5 and MZ‐11 had the highest tolerance to As(V), with EC_50_ values of 1281 and 1108 mg/L, respectively, followed by HXC‐5 (653 mg/L), HLX (475 mg/L), and HG‐2 (421 mg/L). In total, the five target strains MZ‐11, DT‐5, HXC‐5, HLX, and HG‐2 showed stable trends of dry weight decline before 800 mg/L, and the downward trend of strain DT‐5 biomass from 800 to 1200 mg/L became steeper.

As shown in Figure 3, the dry weights of strains PR‐3, MZ‐2, and PR‐2 changed slightly under 0–200 mg/L Cd(II). However, the dry weight of strain PR‐3 decreased in a straight line until it stopped multiplying at 200–800 mg/L Cd(II), and the propagation of strain PR‐2 was completely inhibited at 1200 mg/L Cd ion. The EC_50_ values of strains MZ‐2 and PR‐2 were 1112 and 1104 mg/L, respectively, which were greater than that of strain PR‐3 (407 mg/L), suggesting that both MZ‐2 and PR‐2 had stronger tolerance to Cd(II) stress.

#### Adsorption and accumulation of As and Cd in the mycelium

**FIGURE 4:**
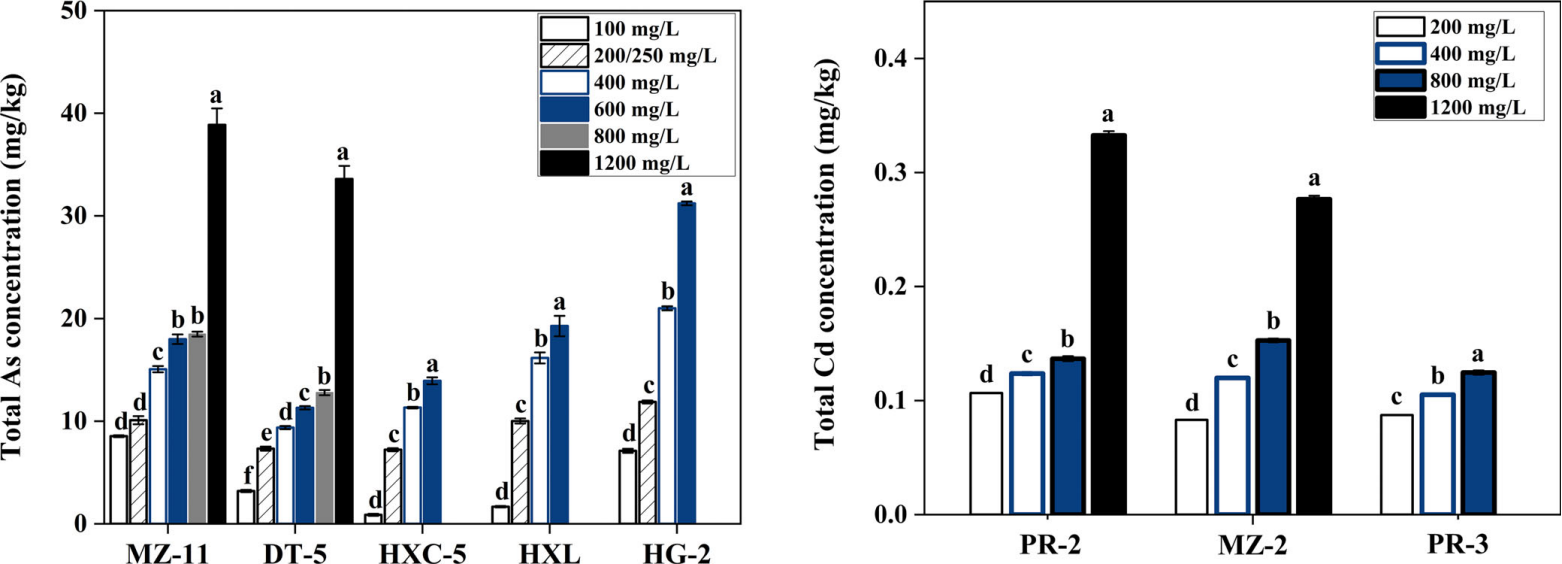
Total As/Cd accumulation in the mycelium of eight dark septate endophyte strains under different As(V)/Cd(II) concentrations. A gradual increase in As/Cd contents in the mycelium of these eight strains under the increase of As/Cd concentrations in the modified Melin‐Norkrans medium. The total As concentration of strains MZ‐11, DT‐5, HXC‐5, HLX, and HG‐2 increased significantly by 3.55‐, 9.52‐, 14.90‐, 10.55‐, and 3.38‐fold with the increasing As(V) concentrations from 100 to 1200 mg/L. The total Cd contents of strains of MZ‐2 and PR‐2 significantly increased by 2.33‐ and 2.12‐fold, respectively, under the Cd(II) concentration from 200 to 1200 mg/L, and were significantly greater than that of strain PR‐3 under each concentration. Different lowercase characters indicate statistically significant differences among treatments for each strain, *p* ≤ 0.05.

A gradual increase in As/Cd content was observed in the mycelia of these eight strains with increasing As/Cd concentrations in the MMN medium (Figure 4). The total As concentration of strains MZ‐11, DT‐5, HXC‐5, HLX, and HG‐2 increased significantly (3.55‐, 9.52‐, 14.90‐, 10.55‐, and 3.38‐fold, respectively) with increasing As(V) concentrations from 100 to 1200 mg/L, and the highest As concentrations in mycelium were 38.87, 33.59, 13.93, 19.27, and 31.22 mg/kg, respectively.

The total Cd contents of strains of MZ‐2 and PR‐2 were significantly greater than that of strain PR‐3 under each Cd(II) concentration, reaching the maximum values of 0.28 and 0.33 mg/kg, respectively, under 1200 mg/L Cd(II). The total Cd content in the mycelium of strain PR‐3 was only 0.12 mg/kg under its maximum Cd(II) treatment concentration of 800 mg/L.

#### Changes in the antioxidant system of target strains under As(V) or Cd(II) stress

**FIGURE 5:**
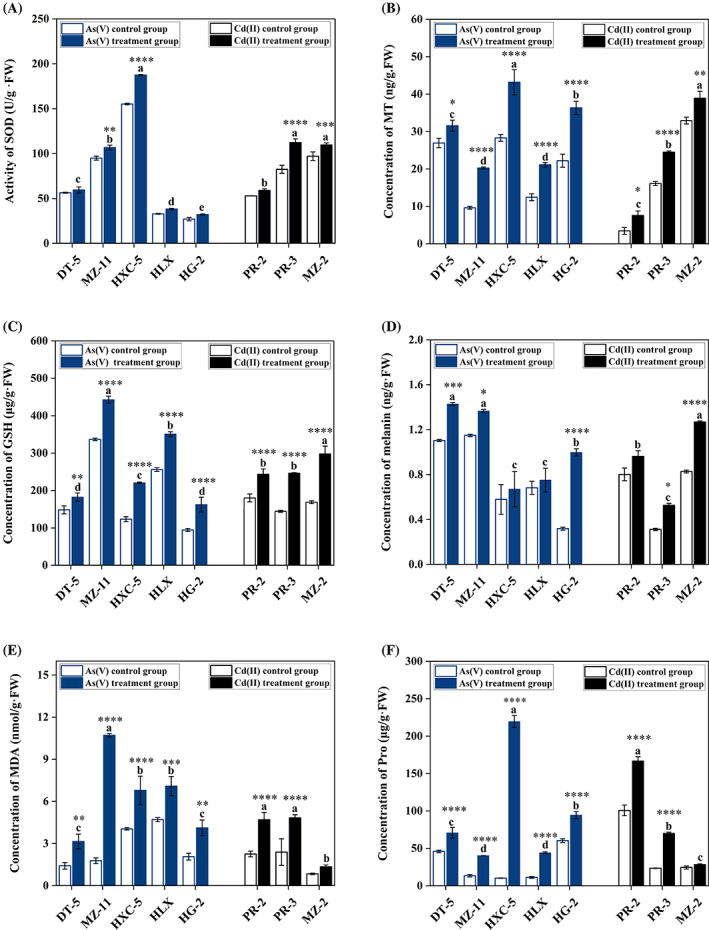
The activity of superoxide dismutase (SOD, A) and the contents of metallothionein (MT, B), glutathione (GSH, C), melanin (D), malondialdehyde (MDA, E), and proline (Pro, F) of the eight dark septate endophyte strains under corresponding EC_50_‐concentration As(V)/Cd(II) of strains. Compared with control groups, SOD activities and contents of the MT, GSH, melanin, MDA, and Pro generally increased under the supplementation with the corresponding EC_50_‐concentration As(V)/Cd(II). Different lowercase characters indicate statistically significant differences among groups under the treatment of As(V) or Cd(II), *p* ≤ 0.05. ‘*’, ‘**’, and ‘***’ indicate the difference between the treatment and its control group at the levels of 0.05, 0.001, and 0.0001.

As shown in Figure 5A, the SOD activities of strains HXC‐5 and MZ‐11 increased significantly by 20.78% (*p* < 0.0001) and 12.44% (*p* < 0.01) under the stress of corresponding EC_50_‐concentration As(V) of strains, respectively, as well as the strains of PR‐3 and MZ‐2 under Cd(II) stress. Compared to the non‐As/Cd treatment groups, the MT and GSH contents of the eight strains significantly increased under supplementation with the corresponding EC_50_‐concentration of As(V)/Cd(II) (Figure 5B,C). Strain MZ‐11 showed the largest increase in MT content with a value of 110.75% under As(V) stress, and strain PR‐2 showed an increase of 130.18% under Cd(II) treatment. The increase in GSH content ranged from 23.18% to 79.23% for the five As‐resistant strains, and the range was 36.12%–76.37% for the three Cd‐resistant fungi.

Strain DT‐5 had the highest melanin content (1.43 ng/g), whereas strain HG‐2 showed the largest increase (214.49%) (Figure 5D). Similarly, exposure to As(V)/Cd(II) significantly enhanced the MDA and Pro concentrations in the eight strains. In the As‐treated groups, the MDA content of strain MZ‐11 increased considerably by 513.10%, and the Pro increase rate of strain HXC‐5 was a staggering 2044.80%, which was in line with the Cd‐resistant strains PR‐2 and PR‐3, which increased by 101.31% and 66.68% (MDA/Pro), and 1111.51% and 198.10% (MDA/Pro), respectively (Figure 5E,F).

#### Phylogenetic analysis

**FIGURE 6:**
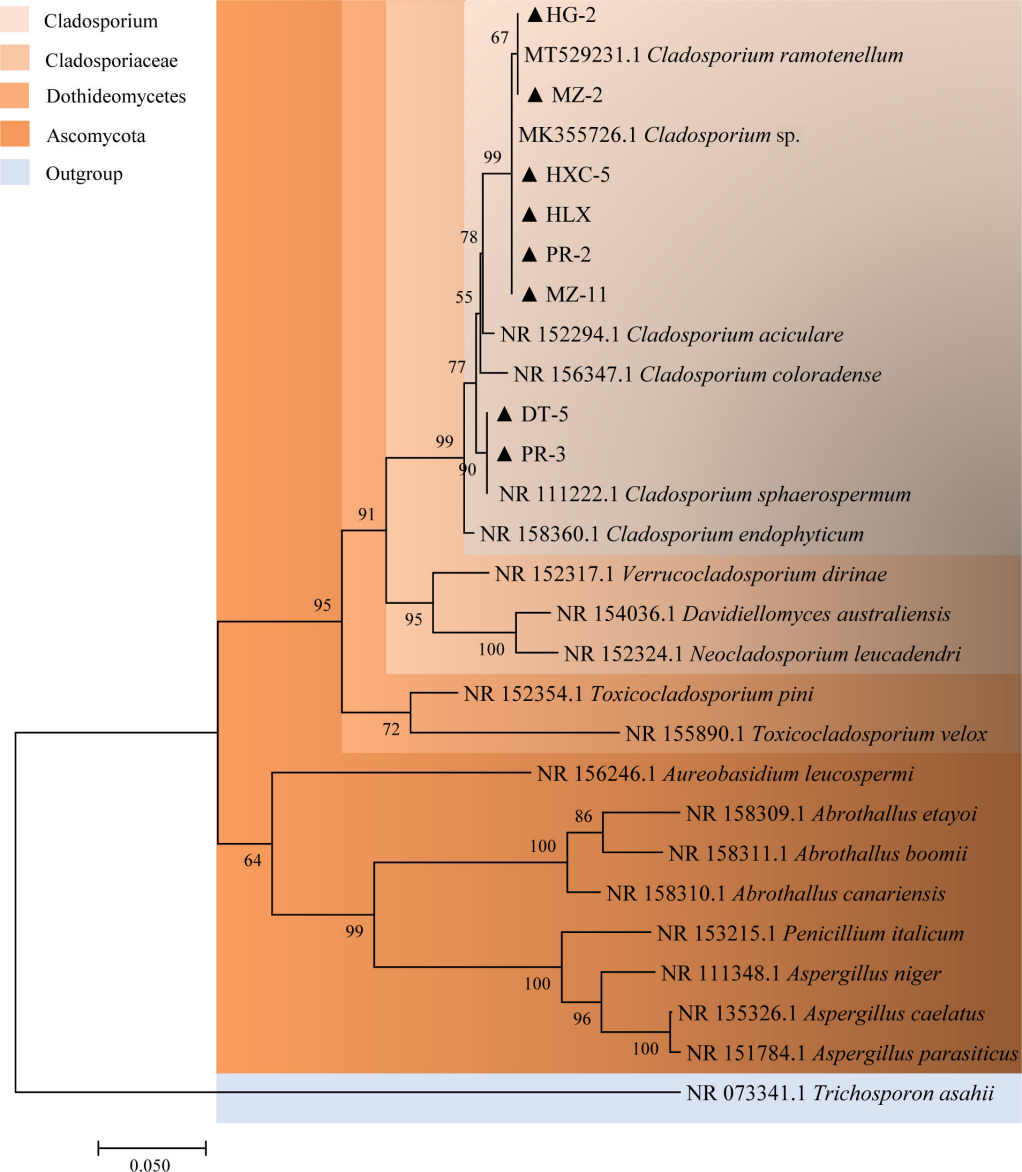
Phylogenetic analysis of the eight dark septate endophyte (DSE) strains. Eight DSE strains were clustered into the clades of *Cladosporium* with a bootstrap value of 99%. Strains of MZ‐11, PR‐2, HXC‐5, and HLX showed high genetic relatedness within the group of *Cladosporium* sp. with a 99% bootstrap value. Strains of MZ‐2 and HG‐2 clustered with *Cladosporium ramotenellumin* as a clade with a 67% bootstrap value, and strains of PR‐3 and DT‐5 were clustered into the *Cladosporium sphaerospermum* clade with 90% bootstrap values. Bootstrap values that are greater than 50% (1000 replicates) are shown above or below the nodes. The scale bar indicates nucleotide substitution in neighbour‐joining analysis. *Trichosporon asahii* was used as an outgroup reference.

As listed in Table 2, the ITS rDNA sequences of eight strains were amplified by PCR and then sequenced; the amplicon sizes were in the range of 510–620 bp. Comparisons using BLAST showed at least 99% homology with other authentic endophytic fungi previously stored in GenBank. To validate the BLAST results, 20 sequences were analysed via ClustalW comparison with the target sequences, after which a phylogenetic tree was generated using the neighbour‐joining method (Figure 6). The tree was rooted in *Trichosporon asahii*. Ascomycota, Dothideomycetes, *Cladosporiaceae*, and *Cladosporium* represented the common phyla, orders, families, and genera of all isolates in the region, respectively. Eight DSE strains were clustered into clades of *Cladosporium* with a bootstrap value of 99%. Strains MZ‐11, PR‐2, HXC‐5, and HLX showed high genetic relatedness within the group of *Cladosporium* sp. with a 99% bootstrap value. Strains MZ‐2 and HG‐2 clustered with *Cladosporium ramotenellumin* as a clade with a 67% bootstrap value, and strains PR‐3 and DT‐5 were clustered into the *Cladosporium sphaerospermum* clade with a 90% bootstrap value. Combined with globose dendrites and other morphological characteristics, *Cladosporium* spp. were confirmed to be the dominant strains in the roots of healthy *P. kingianum* and to play an important role in improving HM tolerance.

**TABLE 2:** Blast search analysis based on ITS rDNA sequences of closest relatives of eight dark septate endophyte strains isolated from *P. kingianum.*

Isolates	GenBank accession number	Close relatives	Identity (%)
MZ‐2	OL818311	*C. angustiterminale*	99.46
MZ‐11	OL818312	*C. welwitschiicola*	100
PR‐3	OL818313	*C. sphaerospermum*	100
PR‐2	OL818314	*C. funiculosum*	100
HG‐2	OL818315	*C. montecillanum*	99.46
HLX	OL818316	*C. phaenocomae*	100
HXC‐5	OL818317	*C. welwitschiicola*	100
DT‐5	OL818318	*C. sphaerospermum*	100

## DISCUSSION

To adapt to the HM stress environment, plants have been found to recruit microorganisms to colonize their roots, which are often characterized by strong HM tolerance, for example, the DB146 strain and *Phialophora mustea* isolated from *Salix caprea* and poplars, respectively (Likar & Regvar, 2013; Yung et al., 2021; Zafar et al., 2007). However, few studies on HM‐tolerated strains from medical plants have been conducted, particularly in *P. kingianum*. To decrease the oxidative damage and toxicity caused by HMs, HM‐resistant strains with clear functions are usually prepared as microbial agents to inoculate the roots of plants planted in HM‐ contaminated areas. It is also one of the most important biological means to reduce HM accumulation in medicinal plants such as *P. kingianum* and *Panax notoginseng*. In our study, eight strains with strong As(V) or Cd(II) tolerance were screened from the fibrous roots of *P. kingianum* that *were* planted in the HM‐contaminated area, the morphological features of which were consistent with the typical structural description of *Cladosporium*, containing diaphragms, typical spores, and spore peduncles (Figure 1, Table 1) (Pelo et al., 2020). In terms of taxonomic status, these eight strains belonged to the DSE, which had typical morphological features of dark mycelia and diaphragmatic hyphae. The phylogenetic tree results further confirmed that these eight DSE strains clustered into a branch of the genus *Cladosporium* with three distinct species: *Cladosporium* sp., *C. ramotenellumin*, and *C. sphaerospermum* (Figure 6). Among filamentous fungi, *Cladosporium* spp. have been widely investigated in numerous studies on the diversity of plant endophytic fungi, such as *Solanum tuberosum* (Bensaci et al., 2022), *Vicia faba* (El‐Dawy et al., 2021), *Glycine max* (Shi et al., 2020), *Triticum aestivum* (Zhang et al., 2022), and *Coptis chinensis* (Song et al., 2018). Increasing evidence has shown that *Cladosporium* are the major root endophytic fungi, but not phytopathogens (Văcar et al., 2021).

Evidence has shown that the genus *Cladosporium* has a relatively strong broad‐spectrum tolerance to HMs such as Cu, Hg, Al, Au, Ag, Cd, and Fe (Cudowski & Pietryczuk, 2019; Văcar et al., 2021). However, the tolerance of *Cladosporium* to As has rarely been reported. The EC_50_ value is considered to be one of the important indicators to assess the susceptibility of strains to HMs (Shadmani et al., 2021). In this study, *Cladosporium* spp. (strain DT‐5 and MZ‐2) isolated from *P. kingianum* showed strong tolerance to As(V) and Cd(II) with reference EC_50_ values of 1281 and 1112 mg/L, respectively (Figure 3), in which the EC_50_ value of strain MZ‐2 for Cd was significantly higher than that of the *E. pisciphila* strain (332.2 mg/L) (Zhang et al., 2015). Tripathi et al. (2020) reported that half of the investigated soil fungal strains showed high tolerance to As(V), with medium concentrations in the range of 100–1000 mg/L when the survival of surviving fungi was 50%. Similarly, the EC_50_ values of strains MZ‐11, HXC‐5, HLX, and HG‐2 were 1108, 653, 475, and 421 mg/L, respectively, under As stress (Figures 3 and 4). When the As(V) concentration reached 1800 mg/L, the growth of the five strains was severely inhibited. The ability of strains MZ‐11, DT‐5, and HG‐2 to accumulate total As was the same as those reported in previous studies, with an accumulation of total As of 38.86 mg/g for strain MZ‐11, 33.59 mg/kg for strain DT‐5, and 31.22 mg/kg for strain HG‐2 (Figure 4), which was close to the reference value of 21.5 mg/g for *Rhizopus* sp. and *Trichoderma* sp. (Batista et al., 2016).

Under Cd stress, the EC_50_ values of strains MZ‐2 (1112 mg/L) and PR‐2 (1104 mg/L) were remarkably larger than those of *E. pisciphila* (112 mg/L) and *Microdochium bolleyi* (414.46 mg/L) reported by Zhang et al. (2015) and Shadmani et al. (2021). However, the accumulation capacity of strains PR‐2, PR‐3, and MZ‐2 seemed to be weaker than that of the strains reported previously, in which the accumulation levels of Cd were only 0.33, 0.28, and 0.12 mg/kg for strains MZ‐2, PR‐2, and PR‐3, respectively; significantly lower than the reference values of 2.63 and 2.72 mg/g for *Cladosporium* sp. and *Rhizopus* sp., respectively (Mohammadian Fazli et al., 2015; Zafar et al., 2007), inferring that resistant means of strains MZ‐2, PR‐2, and PR‐3 mainly depended on excluding Cd ions.

The regulatory mechanism of *Cladosporium* spp. for As(V)/Cd(II) has rarely been elucidated. However, many reports on the HM tolerance mechanisms of DSE strains can be used as references for this explanation. Increasing evidence has shown that SOD, MT, GSH, melanin, MDA, and Pro can be used to uncover the resistance mechanisms that protect cells from oxidative damage. SOD is capable of maintaining a dynamic balance between oxidative radical production and scavenging in vivo, thereby reducing cellular damage caused by oxidative stress (Vallino et al., 2009). Under Cd/As stress, the SOD activity of *E. pisciphila* and *Aspergillus niger* significantly increased, which aligns with our findings that these eight strains exhibited elevated SOD activity under the corresponding EC_50_‐concentration of As(V)/Cd(II) (Figure 5), particularly strains HXC‐5 and PR‐3. This suggests a positive relationship between SOD and resistance to oxidative damage by As(V)/Cd(II) (Mukherjee et al., 2010; Zhang et al., 2015). Chelation of MT and GSH is a common strategy to detoxify metal ions, mainly by segregating the chelating complexes into different organelles and storing them in a low/nontoxic form (Priyadarshini et al., 2021). However, the role of MT in As toxicity remains unclear (Reddy et al., 2016; Thorsen et al., 2012). *Laccaria bicolor* responded to Cd/As induction, exhibiting a significant increase in GSH, and *Paxillus involutus* responds to Cd stress, with a significant increase in MT content (Bellion et al., 2007; Khullar & Sudhakara Reddy, 2019). We found that these eight strains exhibited a significant upregulation of GSH and MT synthesis under either As or Cd stress, indicating that both MT and GSH respond to As‐induced expression (Figure 5).

Reports have shown that DSE strains can eliminate oxidative free radicals caused by HMs by increasing melanin content to improve HM tolerance (Zhang et al., 2016). However, its effect on HM tolerance is somewhat controversial; Zhang et al. (2016) and Potisek et al. (2021) found that melanin content is not related to HM tolerance. Our results showed that the melanin content of most *Cladosporium* spp. increased significantly under As/Cd stress, particularly in the strain MZ‐11 (Figure 5). MDA and PRO are involved in fungal tolerance to HMs as a class of substances that prevent the entry of exogenous substances into cells, thereby causing cellular oxidative damage during fungal osmotic environmental stress (Dachuan & Jinyu, 2021). The same conclusion was drawn from our experiments, where As/Cd treatment significantly increased both levels in all strains except for MZ‐2, indicating that the latter may have an inherent characteristic of high tolerance to Cd (Figure 5). Similar results have shown that Cd strongly induces the synthesis of GSH in microalgae (Li et al., 2021).

Metal detoxification and tolerance mechanisms of microorganisms often include uptake‐reduced, elimination‐enhanced, and intravesicular chelation with metal‐binding proteins and peptides, of which the process is regulated by a rich and complex population of genes (Guo et al., 2016; Priyadarshini et al., 2021). The expression of some uptake‐ and transport‐related proteins is particularly important for fungal HM detoxification, for example, the ATP‐binding cassette transporter EpABC2.1 of *E. pisciphila*, multidrug, toxic compound extrusion protein MTE1 of *Tricholoma vaccinum* and major promoter superfamily permease C17235 of *Pisolithus albus*, which can confer stronger Cd/Cu/Ni tolerance in transformants of *Saccharomyces cerevisiae* (Ali et al., 2012; Cao et al., 2019; Majorel et al., 2014; Schlunk et al., 2015). However, the molecular mechanisms underlying HM tolerance in fungi remain unclear and will be the focus of future experiments. Genome sequencing and metabolomics analysis are effective for elucidating the molecular mechanisms involved in the evolution of HM tolerance in DSE strains.

## CONCLUSIONS

In total, eight endophytic fungi isolated from *P. kingianum* were proven to be highly tolerant to As(V)/Cd(II), the resistance mechanisms of which were revealed by an increase in SOD activity, changes in GSH, MT, melanin, MDA, and Pro contents, and the accumulation of metal ions in the mycelium. These eight strains were identified as *Cladosporium* spp., which belong to the DSE groups based on their taxonomic status. Taken together, the preliminary elucidation of As/Cd tolerance and physiological response mechanisms of *Cladosporium* spp. makes it possible to apply them in the bioremediation of HM‐contaminated soils and the ecological planting of herbaceous plants.

## AUTHOR CONTRIBUTIONS

**Guan‐Hua Cao:** Conceptualization (supporting); data curation (lead); formal analysis (equal); funding acquisition (supporting); project administration (supporting); validation (supporting); writing – original draft (equal); writing – review and editing (lead). **Xiao‐Gang Li:** Data curation (supporting); formal analysis (equal); investigation (lead); methodology (lead); writing – original draft (equal); writing – review and editing (supporting). **Chen‐Rui Zhang:** Investigation (supporting); methodology (supporting); validation (supporting). **Yi‐Ran Xiong:** Investigation (supporting); methodology (supporting). **Xue Li:** Investigation (supporting); methodology (supporting). **Tong Li:** Investigation (supporting); methodology (supporting). **Sen He:** Conceptualization (lead); data curation (supporting); formal analysis (supporting); funding acquisition (lead); investigation (supporting); project administration (lead); resources (lead); supervision (lead); validation (supporting); writing – original draft (supporting); writing – review and editing (supporting). **Zheng‐Guo Cui:** Conceptualization (supporting); validation (supporting); writing – original draft (supporting). **Jie Yu:** Conceptualization (supporting); funding acquisition (supporting); project administration (equal); supervision (supporting); writing – review and editing (supporting).

## CONFLICT OF INTEREST STATEMENT

The authors declare no conflicts of interest.

## Data Availability

The data that support the findings of this study are available from the corresponding author upon reasonable request.
